# Complete genome and plasmid sequences of a multidrug-resistant Salmonella enterica subsp. enterica serovar Panama isolate from a German cattle farm

**DOI:** 10.7150/jgen.48656

**Published:** 2020-06-29

**Authors:** Anne Busch, Helmut Hotzel, Ulrich Methner

**Affiliations:** 1 Friedrich-Loeffler-Institut, Institute of Bacterial Infections and Zoonoses (IBIZ), Naumburger Str. 96a, 07743 Jena, Germany.; 2 University Hospital Jena, Department of Anaesthesiology and Intensive Care Medicine, Am Klinikum 1, 07747 Jena, Germany.

**Keywords:** sequences, genes, genome

## Abstract

We describe a rare isolate of Salmonella enterica subsp. enterica serovar Panama with an extended-spectrum β-lactamase (ESBL) profile from a German cattle-fattening farm. Applying two next-generation sequencing methods we generated sequences of the genome as well as the plasmids; assembled the draft genome sequence of Salmonella enterica subsp. enterica serovar Panama isolate 18PM0209. Antimicrobial resistance genes, virulence-associated genes and plasmids were analyzed using bioinformatics. Occurrence of multidrug-resistant *Salmonella* serovars at cattle-fattening farms indicate the need of enhanced surveillance to prevent further spread of these organisms.

## Introduction

Salmonella enterica subsp. enterica serovar Panama (*S.* Panama) was first isolated during an analysis of a food-borne infection in Panama in 1934 1. The serovar Panama is rarely discussed but has a strong association with invasive diseases and is linked with infections in humans 2. S. Panama is known to cause a significant proportion of *Salmonella* infections in the pig industry 3. This serovar has also been isolated from reptiles and environmental reservoirs, rarely from cattle but is in general considered as understudied 2, 4. *S.* Panama isolates from various origin exhibit different levels of antimicrobial resistance, however, multidrug-resistant isolates may occur 5. A large repertoire of transmission modes is associated with antibiotic resistance. It is suspected that high antibiotic selective pressure in humans or food-producing animals results in antibiotic resistance via the acquisition of numerous types of plasmids or mobile genetic elements 6-8. Here, we present an analysis of genomic and plasmid sequences of S. Panama 18PM0209 characterized by a multidrug-resistant extended-spectrum β-lactamase (ESBL)-producing phenotype isolated from a cattle-fattening farm in Germany in 2018. We analyzed the isolate for genomic features such as antibiotic resistance genes, virulence-associated genes and plasmid replicons.

## Material and methods

Salmonella enterica subsp. enterica serovar Panama (isolate 18PM0209, Panama 1,9,12:l,v:1,5) and isolates from *Salmonella* serovars Hadar (18PM0219 and 18PM0220) as well as Paratyphi B (18PM0210 and 18PM0222) were isolated at the same cattle farm and in the same period. Analysis of fecal samples was carried out according to ISO 6579-1 9, *Salmonella* isolates were serotyped using poly- and monovalent anti-O as well as anti-H sera (SIFIN, Diagnostics GmbH, Berlin, Germany) according to the Kauffmann-White scheme.

Antimicrobial susceptibility of the isolates was assessed by determining the minimum inhibitory concentration (MIC) using the broth microdilution method with Sensititre EUVSEC plates (Trek Diagnostic Systems Ltd., East Grinstead, United Kingdom). Epidemiological cut-off values were used according to the European Committee on Antimicrobial Susceptibility Testing (EUCAST) 10.

Plasmids were prepared with QIAGEN® Plasmid Mini Kit (QIAGEN GmbH, Hilden, Germany) according to the manufacturer's instructions and separated by agarose gel electrophoresis 11.

For whole-genome sequencing, the isolates were grown overnight at 37°C in 3 ml of Luria-Bertani broth (SIFIN, Diagnostics GmbH). Genomic DNA was prepared according to the Nextera™ DNA Flex Microbial Colony Extraction Protocol 12. The plasmids were isolated from an overnight culture with QIAGEN Plasmid Mini Kit. Fifteen microliter of the eluted DNA were separated in an agarose gel (0.8 %, ethidium bromide-stained). Bands were excised and frozen for 30 min at -80°C, centrifuged at 14,000 rpm for 1 min, and the supernatant was used for the library preparation with a Nextera Flex and NexteraXT DNA Library Preparation Kit (illumina, San Diego, USA). Whole-genome sequencing libraries were constructed using the Nextera Flex and NexteraXT DNA Preparation Kit according to manufacturer instructions. Paired-end sequencing was performed on the Illumina MiSeq platform (illumina Inc.) using a 300-cycle MiSeq reagent kit. A quality check was performed with FastQC. Reads were *de novo* assembled using SPAdes 3.12.0 in Bayes Hammer mode (--careful) 13 and evaluated with QUAST v4.3 14 in standard setting. The filtering of the sample was performed by removing contigs with coverage less than 5× and size below 500 bases as described before 15. ResFinder 16, PlasmidFinder 17, SeqSero 18, SPIFinder 19 and its databases were accessed through the Center of Genomic Epidemiology (http://www.genomicepidemiology.org/, accessed January 2020).

## Results

**Figure 1:**
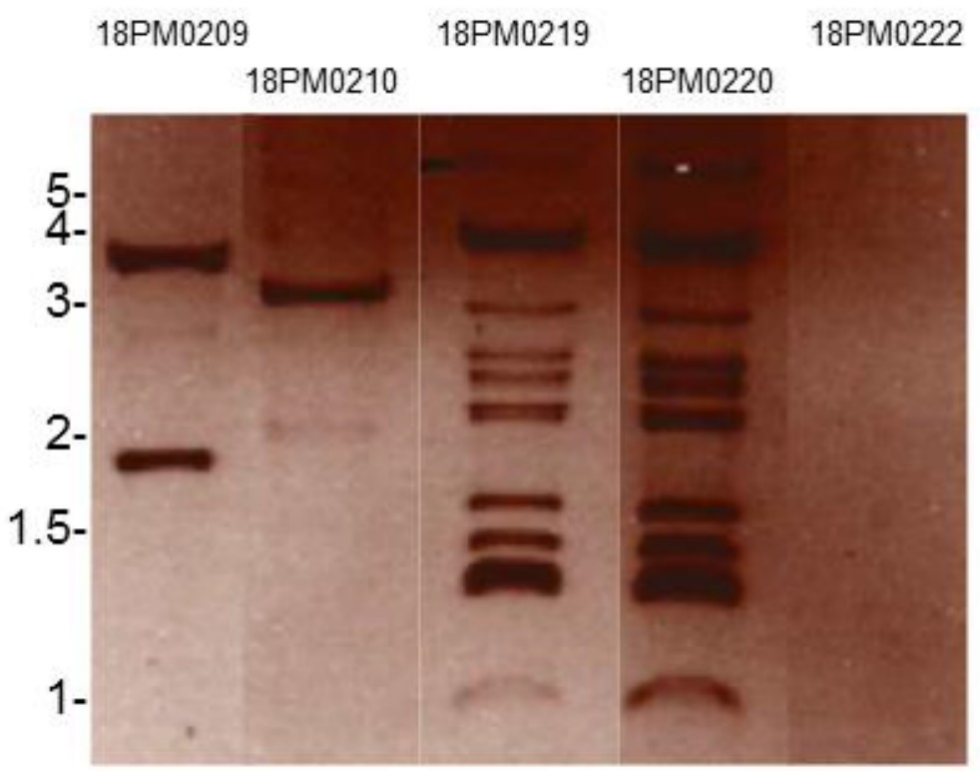
Plasmid preparation of *Salmonella* isolates separated by agarose gel electrophoresis: 18PM0209 (Panama), 18PM0210 (Paratyphi B), 18PM0219 (Hadar), 18PM0220 (Hadar), 18PM0222 (Paratyphi B) (The marker is given in kb).

Salmonella enterica subsp. enterica serovar Panama 18PM0209 and 4 *Salmonella* isolates belonging to other serovars from the same year and the same farm were studied. To analyze horizontal gene transfer with plasmids within the species, we isolated plasmids and tested the isolates for antibiotic resistance. All isolates carried different plasmids regarding number and size (Figure 1).

Salmonella enterica subsp. enterica serovar Panama 18PM0209 had two plasmids. Isolate 18PM0210 of *S.* Paratyphi B had one plasmid of 3.5 kb. The isolates 18PM0219 and 18PM0220, both *S.* Hadar, harbored each plasmids from 1 kb to 4 kb, but should be considered with caution as extra bands can occur when plasmid DNA is nicked, linearized and/ or permanently denatured. The isolate 18PM0222 of *S.* Paratyphi B had no plasmid.

This was reinforced with analysis of antibiotic resistances. Within the serovars, antibiotic resistance pattern varied greatly, however, only *S*. Panama revealed resistance to cefotaxime, ceftazidime (typical for an ESBL isolate) and gentamicin (Table 1).

The whole-genome assembly for Salmonella enterica subsp. enterica serovar Panama 18PM0209 (chromosome and plasmids) was represented by 2,408 contigs with an N50 contig length of 3,295 bp, in which the largest contig had 372,036 bp. The sequence length of the contigs was 4,755,767 bp with a GC content of 51.7%.

A conclusive analysis with bioinformatics started with ResFinder, predicting an acquired antimicrobial resistance to aminoglycoside as gentamicin. Aminoglycoside antibiotic was tested *in vivo* and showed a high antibiotic resistance (Table 1). Additional resistance to the following antibiotics was detected: tetracycline, oxazolidinone, sulphonamide, aminoglycoside, nitroimidazole, fusidicacid, phenicol, rifampicin, β-lactam, fosfomycin, macrolide, trimethoprim, colistin, glycopeptide, quinolone (aminoglycoside: aac(6')-Iaa: NC_003197; aph(3'')-Ib (aph(3'')-Ib): AF321551; aph(3')-Ia: EF015636; aph(6)-Id (aph(6)-Id): M28829. β-lactam: blaTEM-1A (RblaTEM-1): HM749966. phenicol: floR: AF118107. quinolone: qnrB19 (with warnings): EU432277). As the isolate is highly resistant against ciprofloxacin, we analyzed *gyrAB* or parC genes (*gyrA*: 11824019, *gyrB*: 6924602, *parC*: 10969692, *gyrA*: 44981070). These had sequence identity of 82.9 % to 97.8 %, and were to mapped with the plasmid derived reads, only with the whole genome reads, indication for an possible chromosomal resistance mechanism.

Serotyping with traditional methods as described before resulted in the serovar Panama. The bioinformatics prediction with SeqSero-1.2 prediction the serovar was falsely classified as Gallinarum. The O antigen was identified as O-9. The H antigen was not identified and thus the serotype Panama was not identified correctly. Mistyping with SeqSero1.2 is common as reported previously 20.

The following *Salmonella* Pathogenicity Islands were identified with the SPI Finder 19: SPI-13 (AY956834), SPI-13 (AY956832), SPI-14 (AY956836), SPI-13 (AY956833) and C63PI (AF128999). Other SPI-correlated genes could be mapped indicating at least the pathogenicity island 1 but not with the plasmid specific reads (*hilA* - 6923823, *orf319* - 6924315, *pipA* - 6925394, *pipB* - 6924440, *prgH* - 6924497, *prgI* - 6924498, *prgJ* - 6924499, *prgK* - 6922650, *SG1706* - 6924926, *SG2315* - 6923734, *SG2806* - 6922652, *sipA* - 6923921, *sipB* - 6924853, *sipC* - 6924854, *sipD* - 6924851, *ssaB* - 6924919, *ssaD* - 6924921, *ssaG* - 6924923, *ssaH* - 6924924, *ssaI* - 6924925, *ssaJ* - 6922416, *ssaK* - 6924927, *ssaM* - 6924937, *ssaU* - 6925518, *ssaV* - 6924929, sscB - 6924948, *sseA* - 6924943, *sseB* - 6924939, *sseC* - 6924946, *sseD* - 6924947, *sseE* - 6924940, *sseF* - 6924949, *sseG* - 6924922).

PlasmidFinder 2.0 17 identified the following plasmid replicon sequences: Col (pHAD28) (100 %, KU674895), IncHI2 (100 %, BX664015), IncHI2A (99.39 %, BX664015), IncI2 (Delta) (98.14 %, AP002527). Two plasmids could be detected and sequenced in 18PM0209 (4kb and 7kb). The 4kb plasmid had 76 % identity to the plasmid R478 of *Serratia marcescens*, a bacterium belonging to the genus *Serratia*, the family *Yersiniaceae* and the order of *Enterobacterales.* On a length of 75 kb of the reference AP002527 47 % were identical with a mean coverage of 131 (StdDev 30 reads). The 7 kb plasmid had 76 % identity to the plasmid R721 of *Escherichia (E.) coli*.

To evaluate the sequencing quality of the two techniques we compared the reads of NexteraXT library preparation and the reads of the Nextera Flex were mapped on the closest neighbor, reference CP012346. The reference had a 96.9 % identity. With NexteraXT library preparation the mean was 141 (StdDev 28) with a minimum of 0 and a maximum 634. With Nextera Flex library preparation the mean was 133 (StdDev 29) with a minimum of 35 and a maximum 151. In summary the quality of Nextera Flex was better and the evenness of read distribution providing better data, demonstrating a quick and precise method.

## Discussion

Genome sequencing directly from a colony is well established with species from family *Enterobacteriaceae, Klebsiella pneumoniae* and *E. coli* and for example *Pseudomonas aeruginosa* 12. Without adaption, the protocol was successfully applied to *Salmonella* including plasmid DNA with a quick and efficient protocol 12, 21. This easy to handle protocol is applicable for all plasmid preparations.

Salmonella enterica subsp. Enterica serovar Panama 18PM0209, detected at a cattle-fattening farm revealed both a rare serotype in cattle and a multidrug-resistant phenotype. It is important to monitor bacteria with multiple antibiotic resistances to avoid spread within farm animals as well as to prevent these organisms from entering the food chain.

Recombination and rearrangement events in mobile elements are described as driving factors for the evolution and spread of antibiotic resistances. The origin of the extended spectrum β-lactamase profile in *S.* Panama 18PM0209 can be hypothesized only but might be caused horizontally by plasmid transfer. Plasmids as mobile elements are highly prevalent in *Enterobacteriaceae* and are known to carry various resistance determinants including extended spectrum β-lactamase (ESBL) profile and carbapenemases-like KPC 22. There are outbreak reports worldwide on the horizontal transfer of plasmids across different bacterial genera, but the own species is preferred 23. Therefore, we analyzed further *Salmonella* organisms detected from the relevant farm in the same year. The analysis demonstrated that all five isolates harbored plasmids different in numbers and sizes. Therefore, a plasmid-mediated outbreak scenario within these isolates could be excluded. The analysis of the plasmid sequences revealed the existence of IncI indicator sequences that are most typical low copy plasmids with conjugative transfers predominant in E. coli and S. enterica serovars detected in Europe. They are also typical for poultry but rather atypical for cattle farms 24. The serovar *S.* Panama is reported to be most susceptible to antibiotics in Germany (except for streptomycin) but can be multi-resistant as reported from Asia and South America 2, 5, 25, 26. We present here a new rare clone of this serovar with an acquired antibiotic resistance.

Antibiotic resistance genes are frequently located on mobile elements as conjugative plasmids or conjugative transposons, but also on bacteriophages and can be transferred by transformation 27. In cattle-fattening farms, the regular and constant entry of calves from different other cattle farms is linked with a permanent introduction of a wide range of microbiota of most different composition and characteristics. Therefore, keeping of animals under conditions of “crowding disease” enables with high probability a rather rapid and extensive exchange of genetic components between different bacterial species from the same or different genera. Whether any prophylactic or therapeutic antibiotic treatment at these farms might also affect the development of multidrug-resistant organisms can only be suspected. In *S.* Panama plasmids of 3 to 20 kb are reported and they can be responsible for full virulence 28. The virulence analysis identified 14 *Salmonella* Pathogenicity Islands. The identified SPIs were typical for *S.* Gallinarum and few in numbers (1-14 SPI, 29).

In summary the quality of NexteraFlex library from illumina was excellent. It provided more evenly distributed data than the NexteraXT library, also both methods are transposase based, as reported before 30. This demonstrates a quick and precise method, also to sequence plasmids and is applicable with free in the internet available Salmonella specific bioinformatics methods.

In summary, the results indicate that the plasmids characterized in *S.* Panama 18PM0209 may be acquired either from other *Salmonella* organisms or even different genera like Escherichia or Serratia at the cattle farm. Whether the use of antibiotics at cattle-fattening farms might also be a risk factor for the development of multidrug-resistant *Salmonella* and other *Enterobacteriaceae* can neither be excluded nor confirmed. However, enhanced surveillance to prevent further spread of this multidrug-resistant Salmonella Panama in cattle farms is recommended - best with the easy to handle method proposed here.

## Nucleotide sequence accession number

Whole genome for Salmonella enterica subsp. enterica serovar Panama isolate 18PM0209 was submitted in the BioProject PRJNA602590, and BioSample SAMN13899429. The version described in this paper is the first version.

## Figures and Tables

**Table 1:** ** MIC values (μg/ml) of antimicrobial substances in *Salmonella* isolates *in vivo.***(SMX= sulfamethoxazole, TMP= trimethoprim, CIP= ciprofloxacin, TET= tetracycline, MERO= meropenem, AZI= azithromycin, NAL= nalidixic acid, FOT= cefotaxime, CHL= chloramphenicol, TGC= tigecycline, TAZ= ceftazidime, COL= colistin, AMP= ampicillin, GEN= gentamicin**).**

Antimicrobial substances	*S.* Panama 18PM0209	*S.* Paratyphi B 18PM0210	*S.* Hadar 18PM0219	*S.* Hadar 18PM0220	*S.* Paratyphi B 18PM0222
SMX	> 1024	> 1024	> 1024	> 1024	> 1024
TMP	2	> 32	0.5	0.5	> 32
CIP	8	2	2	1	1
TET	> 64	8	> 64	> 64	8
MERO	0.12	0.06	0.06	0.06	0.06
AZI	64	64	32	16	32
NAL	> 128	> 128	64	64	> 128
FOT	2	0.5	< 0.25	< 0.25	0.5
CHL	> 128	16	16	16	16
TGC	8	2	2	2	2
TAZ	4	2	2	1	2
COL	2	< 1	< 1	< 1	< 1
AMP	> 64	> 64	> 64	> 64	4
GEN	> 32	1	2	2	1
